# Point-of-Care Diagnostics: Recent Advances and Trends

**DOI:** 10.3390/bios7040062

**Published:** 2017-12-18

**Authors:** Sandeep Kumar Vashist

**Affiliations:** IDS Immunodiagnostic Systems Deutschland GmbH, Rahmhofstr. 2-4, 60313 Frankfurt am Main, Germany; sandeep.vashist@idsplc.com; Tel.: +49-157-8827-8723

**Keywords:** point-of-care, diagnostics, biosensors, assays, devices, modeling, framework, bioanalytical applications

## Abstract

Recent years have witnessed tremendous advances in point-of-care diagnostics (POCD), which are a result of continuous developments in biosensors, microfluidic, bioanalytical platforms, assay formats, lab-on-a-chip technologies, and complementary technologies. This special issue targets the critical advances in POCD and provides guided insights and directions for future research.

Point-of-care testing (POCT) is essential for the rapid detection of analytes near to the patient, which facilitates better disease diagnosis, monitoring, and management. It enables quick medical decisions, as the diseases can be diagnosed at a very early stage, leading to improved health outcomes for patients by enabling the early start of treatment. The global POCT market is expected to grow from US$ 23.16 in 2016 to US$ 36.96 billion in 2021 at the compound annual growth rate (CAGR) of 9.8% from 2016 to 2021 [1]. North America accounts for most of the global POCT market, followed by Europe, while Asia-Pacific’s POCT market is expected to grow the most at the CAGR of 14.2% [1].

Several prospective POCD have been developed during recent years, which are paving the way to next-generation POCT [2]. The biosensor is the most critical component of POCD, and is directly responsible for the bioanalytical performance of an assay. Several prospective label-free biosensors, such as electrochemical, surface plasmon resonance (SPR), white light reflectance spectroscopy (WLRS), etc., have been developed, and are being used for improved POCD. Complementary technologies, e.g., microfluidics, lab-on-a-chip technologies, system integration, device automation, and signal readout, are providing the desired impetus for continuous improvements in POCD.

Harpaz et al. [3] provided a comprehensive review of POCT in acute stroke management, which emphasizes the unmet need for desired POCT technologies, and quantitative and multiplex POCT platforms. POCT plays an important role in patient management, as it critically shortens the time-to-treatment, enables classification of stroke subtypes, and improves the patient’s outcome. A wide range of point-of-care (POC) assays for the quantitative determination of biomarkers has been developed using portable and easy-to-use POC clinical and biochemical analyzers. Therefore, the POCT would enable rapid clinical decision-making in the diagnosis of ischemic stroke, which would considerably improve patient outcome by facilitating treatment and medical intervention at an early stage. As mentioned by the authors, there is an upcoming trend for the integration of POCT into established clinical practice. There is an emerging need for the development of next-generation POCT devices that could resolve the gaps in stroke clinical practice by multiplex detection of stroke biomarkers.

On the other hand, Soraya et al. [4] developed an interdigitated electrode impedance spectroscopy biosensor-based POCT for the quantitative detection of fecal hemoglobin in low-resource settings with a limit of detection (LOD) of 10 µg hemoglobin per gram of feces. The developed test has an advantage over conventional fecal occult blood tests, as it doesn’t require any dietary or drug restrictions. Additionally, an interesting amperometry-based immunodiagnostic method was demonstrated by Waller et al. [5] for the detection of *Bacillus anthracis* spores using immunomagnetic separation for the capture of the spores, and a sandwich immunoassay format. The developed method has a short sample-to-answer time of less than one hour, as well as high analytical sensitivity, with the ability to detect down to 500 target spores (5000 cfu mL^−1^). It is based on the detection of spores by polyclonal *B. anthracis* capture of antibody-conjugated magnetic beads and monoclonal *B. anthracis* detection of antibodies bound to glucose oxidase. The glucose oxidase activity of the spores is measured by amperometry. The assay showed high specificity for *B. anthracis* spores, and was unaffected by environmental interferents such as those present in soil. In another study, an interesting computational fluid dynamics (CFD) techniques-based modeling of chamber filling in a micro-biosensor for protein detection was shown by Islamov et al. [6] employing the ANSYS-CFX platform.

Koukouvinos et al. [7] provided a critical review of the WLRS-based label-free sensing platform for the detection of high or low molecular weight analytes. The various bioanalytical parameters, optical set-ups, and performance characteristics of WLRS were discussed by the authors, together with the various interesting biosensing applications and directions for future research.

Of interest is the innovative SPR device reported by Patel et al. [8], which is highly cost-effective, as the cost of the SPR chip used in the system is more than 50-fold cheaper than that used by the BiaCore SPR instruments from GE Healthcare. The cost of the developed SPR device is also many-fold cheaper than that of the BiaCore systems. The authors showed remarkable sensitivity for the detection of botulinum neurotoxin type A chain, with a LOD of 6.76 pg mL^−1^. The detection sensitivity of the developed assay is comparable to that of the conventional gold standard assay, i.e., mouse bioassay, which takes several days to obtain the results. An innovative approach was shown by Mishra and Vazquez [9], who demonstrated the Gal-MµS microfluidics device for the evaluation of cell migratory response to individual and combined Galvano-chemotactic fields, which has only been partly understood, to date. The real-time imaging within the device captures the cell trajectories in response to electrical fields and chemical gradients. The data obtained in the study shows that neural cells migrate longer distances with higher velocities in response to combined stimuli than to individual stimuli. The device might lead to the development of migration-targeted treatments for the improvement of cell-based regenerative therapies in the nervous system.

In another study, Elgendi [10] demonstrated the two event-related moving averages (TERMA) framework for biomedical signal analysis. It is a simple and efficient event detector that could have potential applications in wearable and POC devices. The TERMA framework consists of six independent LEGO building bricks, which enables the detection of events in biomedical signals by monitoring event-related moving averages. The developed generic framework could be employed for the detection of various types of events in biomedical signals.

Torabian et al. [11] demonstrated the improvement in long-term stability of paper-based POC tests for sickle cell anemia by substituting sodium hydrosulfite with sodium metabisulfite. The test showed 24 weeks of shelf stability at room temperature. Moreover, it has superior LOD (10% sickle hemoglobin), reduced sample-to-analysis time (21 min), higher sensitivity and specificity (97.3% and 99.5%, respectively), and increased cost-effectiveness (just 0.21 USD).

Of interest is the comprehensive review by Sanginario et al. [12], which provides insights on the use of carbon nanotubes (CNT) for the diagnosis and treatment of cancer. The roles of CNT for drug delivery [13], cancer imaging, and physical ablation of metastasis have been discussed, together with challenges and limitations such as the biocompatibility and cytotoxicity of CNTs [14].

The current trend in POCD is inclined strongly towards smart devices equipped with mobile healthcare (mH) [15], which could truly revolutionize personalized healthcare monitoring and management, thereby paving the way for next-generation POCT [2]. A wide range of mH technologies have already been developed, the most promising being cellphone-based POC technologies for the readout of colorimetric, fluorescent, chemiluminescent, electrochemical, lateral flow, and label-free assays; detection of cells, biomolecules, nanoparticles, and microorganisms; and other diagnostic applications [16,17]. The number of cellphone users has already passed 7.4 billion, 70% of which are in developing countries, where there is a critical need for POCD. Several cellphone-based devices and smart applications have been commercialized for the monitoring and management of basic health parameters, such as blood glucose, blood pressure, weight, body analysis, pulse rate, electrocardiogram, and physical activity. However, the security and privacy of personal data is a critical concern for mH, in addition to the requirement for establishing international cloud-computing standards and the management of ‘Big Data’. The global efforts will effectively tackle these challenges in the coming years. Moreover, the development in complementary technologies could lead to the development of next-generation POCD. The day is not far off when POCD would be used by most people, which would empower them to monitor and manage their own health.
